# The Registration Campaign

**Published:** 1908-10-31

**Authors:** 


### Nursing Administration.

### THE REGISTRATION CAMPAIGN.

The recent discussion in Committee in the House of Lords on the Nurses' Registration Bill goes far to explain the strong feeling which has been evoked against this measure in responsible quarters. It is well known that an overwhelming majority of those women who are closely connected with the practical work of training nurses are opposed to the principle of registration. They not only dissent from the system of registration now proposed to be established by law, they have also held aloof from the voluntary system of registration inaugurated by the Royal British Nurses' Association. In forming this settled opinion against registration for nurses it is evident that they have been guided by their experience of the conditions under which nurses at present carry on their work. None have better opportunities, it will be admitted, than the heads of large training schools for forming a correct judgment on this matter. Many of them control influential private nursing institutes, and thus supply nurses to the public, in addition to training them. Even where this is not the case matrons keep in touch with former members of the staff, and have continual evidence of the factors which make for success in their careers. If it were true, as is constantly affirmed by the advocates of registration, that nurses have no proper status, are continually mistaken for blundering impostors, and are impeded in the exercise of their profession for want of official recognition, these facts would be common property, and every nurse, whether matron or no, would be enthusiastically in favour of legislation. But in ninety-nine important training centres out of a hundred the exact contrary is upheld. The matrons are emphatically of opinion that registration, in this country at any rate, is not a measure to promote the well-being of nurses or ensure their efficiency. We are not concerned at this moment to reiterate the reasons which influence them against registration. It is sufficient that these reasons exist, and, in consequence, matrons as a body have abstained from belonging to the Society known as "The Matrons' Council of Great Britain and Ireland," and from the kindred Society for the State Registration of Nurses. The members of these Societies are entitled to urge their opinions in public and private meetings, and in the Press, while those who hold aloof are entitled also to the exercise of their faculties, even although they do not meet together to pass resolutions and elect delegates. But it is difficult to justify the position adopted by members of the Matrons' Council with regard to those who dissent from their opinions in this weighty matter of registration. It was open to them to follow the custom of all societies claiming public recognition, to make known the names of their adherents, and to add by degrees to their ranks such matrons as might be drawn over to their side by the cogency of their arguments. In so doing, even if they failed in carrying with them the most influential section of the nursing world, they would have commanded respect. But they have drawn a close veil of secrecy round their members, and in the effort to live up to a name rashly adopted have striven ceaselessly to persuade the public that they are supported generally by the matrons of Great Britain and Ireland. Paucity of numbers is no reproach in itself. The best work of the world has been done by minorities. But pretence in such matters is not only bad in itself, but is such a symptom of hidden weakness as cannot fail to reflect upon the cause. When, in addition to this undesirable concealment, we find accusations levelled against all who differ from the would-be reformers, we are tempted to wonder whether those who use such weapons are not, perhaps, somewhat at a loss for more respectable arguments. It is deeply to be regretted that Lord Ampthill would have lent himself to so grave an imputation against the heads of training schools throughout the kingdom as that put into his mouth by the promoters of the Bill. "The membership of the Matrons' Council might have been much larger but for the fact that many matrons were the paid servants of hospital committees who were strongly opposed to the registration policy of the Council." It is the kind of insinuation which admits of no argument, and can be met with no refutation. Those who are acquainted with the relations of cordial confidence which, with but few exceptions, prevail between the matrons of voluntary hospitals and the governing committees, can only smile at the idea of these capable administrators dissembling their opinions on matters relating to their own department for fear of receiving reprimands or being solemnly cashiered. But the imputation has been made in the assembly of all others where it might have been expected that women of distinguished personal character and abilities, filling arduous posts in the public service with entire devotion, would have been free from aspersion. It will probably be believed in numerous quarters where criticism against hospitals is eagerly caught up. The hospital matrons are cowards, the hospital committees are tyrants, the poor nurses must go on being oppressed unless a benevolent Government will read between the lines and grant the boon for which matrons are secretly sighing, while cringing to their lords and masters.

Is it to be wondered at that this line of conduct has roused a very strong feeling among those who are responsible for nursing education? That it would be much more adequately expressed were the opponents of registration organised into a coherent body is indisputable. There are signs that the *vis inertiæ* hitherto opposed to registration schemes is insufficient. A waiting policy is always distasteful to ardent minds; but it must never be forgotten that precipitate legislation is the strongest of all bars to real reform. It is essential, therefore, that the responsible leaders of nursing in these realms should combine and provide that Parliament shall be brought to see the true bearing of the opposition to nurse registration, that all danger of hurried and ill-considered legislation may be happily removed.

